# Nurses’ knowledge, attitudes, and behaviors of inadvertent perioperative hypothermia prevention in China: a multicenter, cross-sectional study

**DOI:** 10.1186/s12887-025-05920-1

**Published:** 2025-08-08

**Authors:** Lei Yang, Wenjuan Tang, Qingqing Du, Yingmin Liu, Zhen Wang, Xia Yang

**Affiliations:** 1 Department of Orthopedics, Shanghai Childrens’ Hospital, School of Medicine, Shanghai Jiao Tong University, Shanghai, China; 2 Department of Nursing, Shanghai Childrens’ Hospital, School of Medicine, Shanghai Jiao Tong University, Shanghai, China; 3 Department of Operating room, Shanghai Childrens’ Hospital, School of Medicine, Shanghai Jiao Tong University, Shanghai, China

**Keywords:** Nurse, Knowledge, attitudes, and behaviors, Inadvertent perioperative hypothermia, Influencing factors, Multicenter cross-sectional study

## Abstract

**Background:**

Inadvertent perioperative hypothermia (IPH) is a clinical phenomenon in which patient’s core body temperature is below 36℃ due to non-medical purposes during the perioperative period. Children are more s usceptible to hypothermia due to their significantly higher surface area-to-body weight ratio compared to adults, the immaturity of their thermoregulatory centers, and the thinness of their subcutaneous fat insulation layer. Perioperative nursing is an essential component of the multiprofessional management of surgical patients. Since nurses are the primary caregivers and monitors of patients during perioperative process, a better understanding of nurses’ knowledge, attitudes and behaviors regarding IPH prevention is critical to improving patient outcomes. Consequently, a multicenter cross-sectional study was carried out to investigate the current situation of anesthesia and operating room nurses in China, as well as the prevention of inadvertent perioperative hypothermia and its influencing factors.

**Methods:**

In this study, 292 nurses from 7 children’s specialized hospitals in 4 provinces (Hunan, Anhui, Zhejiang, and Shanghai) in the southern, northern, and central regions of mainland China participated in two-stage purposive and convenience mixed sample technique. General demographic information questionnaire, the knowledge, attitudes, and behaviors questionnaire, and the self-efficacy scale were used for data collection. Descriptive analysis, univariate analysis, correlation analysis, and multiple linear regression analysis were used for data analysis.

**Results:**

The results indicated that mean (SD) score of knowledge, attitudes, and behaviors scales were 15.59 (3.28), 47.02 (8.05), and 52.48 (6.35), respectively. Results of multiple linear regression analysis showed that educational level (*t* = 2.278, *P* < 0.05), No. of IPH training (*t* = 3.408, *P* < 0.01) and whether there was a perioperative insulation process (*t*=-3.091, *P* < 0.01) were risk factors for knowledge; No. of IPH training (*t* = 3.175 *P* < 0.01) was risk factor for attitudes; No. of IPH training (*t* = 2.476, *P* < 0.05), whether there was a perioperative insulation process (*t*=-6.612, *P* < 0.01) and self-efficacy (*t* = 2.851, *P* < 0.01) were risk factors for behaviors. Correlation analysis displayed self-efficacy was positively related to knowledge (*r* = 0.137, *P* < 0.05), attitudes (*r* = 0.115, *P* < 0.05), and behaviors (*r* = 0.258, *P* < 0.01); knowledge was also positively correlated with attitudes (*r* = 0.262, *P* < 0.01) and behaviors (*r* = 0.322, *P* < 0.01); attitudes were positively related to behaviors (*r* = 0.153, *P* < 0.01).

**Conclusions:**

To improve knowledge, attitudes, and behaviors of IPH prevention among operating room nurses and anesthesia nurses in children’s specialized hospitals, nursing managers should increase the frequency of IPH training and develop standardized perioperative insulation process. Nurses should also enhance their theoretical knowledge learning, improve educational level and self-efficacy.

## Introduction

Inadvertent perioperative hypothermia (IPH) is characterized by an unplanned reduction in body temperature, harmful to the organism, with a core temperature below 36 ℃, occurring at any point during the perioperative period [1, 2]. In 1988, the American Peri-Operative Registered Nurses (AORN) Association first underscored the necessity to enhance perioperative temperature management for surgical patients [3]. Despite this, over the past 35 years, both domestic and international scholars have demonstrated that the incidence of postoperative hypothermia ranges from 4–72% [4–8], with some studies even reporting rates as high as 90% [9]– [10]. IPH can result in severe complications for surgical patients, including prolonged hospitalization [11], increased risk of pressure ulcers [12], abnormal cardiac function, and even death [13]. Furthermore, it contributes to an increased economic burden on patients and the consumption of healthcare resources. A study conducted in Australia estimated the cost of unintended postoperative hypothermia to be approximately $1,259,725,856 [14]. However, the significance of unintentional hypothermia for patients and community has not received the attention it deserves, and effective and consistent measures to address it are lacking in clinical perioperative practice [15, 16].

The safety and quality of treatment for children undergoing surgery are continual focal points in the perioperative clinical setting. Pediatric patients are more susceptible to hypothermia, due to their relatively larger surface area-to-body weight ratios, diminished thermoregulatory ability, and limited subcutaneous fat [17, 18]. Unintentional hypothermia in pediatric patients can lead to surgical complications such as surgical site infections, increased oxygen requirement, altered pharmacokinetics of drugs, impaired coagulation, and cardiac arrhythmias [19, 20]. Unfortunately, majority of pediatric studies only document the occurrence of postoperative hypothermia, despite intraoperative hypothermia being more prevalent. The results of several studies indicate that the incidence of perioperative hypothermia in children, including neonates and infants, ranges from 45–85% [21–24]. However, other studies demonstrated that even in premature newborns, hypothermia rates can be as low as 10% when appropriate procedures are followed [25–27].

Perioperative nursing is a crucial component of the multiprofessional management of surgical patients. To mitigate the risk of postoperative complications, nurses should employ interventions such as passive warming, active warming, warming irrigation, intravenous fluids, warming anesthetic gases, perioperative pharmacologic vasodilation, and prewarming prior to anesthesia to prevent perioperative hypothermia [28]. Perioperative assessment is vital to identify individuals at risk, enabling nurses to significantly reduce heat loss, lower the risk of related problems, and ultimately enhance short- and long-term patient rehabilitation through the implementation of simple safeguards. It is imperative to minimize skin exposure, provide adequate bed linens in the operating room, and educate patients on the importance of maintaining their body temperature during surgery. Additionally, the use of forced air warming before surgery and active warming may help avoid hypothermia throughout the perioperative period [29]. As primary caregivers and monitors during the perioperative process, a deeper understanding of nurses’ knowledge, attitudes, and behaviors regarding IPH prevention is critical for improving patient outcomes. A study conducted in Gambia revealed that majority of nurses exhibited a high level of awareness/knowledge but a low mean score in practice [29]. However, the findings of a study by Ireland et al. on nurses working at a single trauma unit in Australia indicated that nurses had limited knowledge regarding perioperative hypothermia [30]. A qualitative study of nurse anesthetists and operating room nurses demonstrated that perioperative temperature measurement and measures to prevent IPH were not consistently implemented [31]. Nevertheless, the efficacy of prophylaxis against IPH is well-established.

Review of literatures found that there are many influences on nurses’ IPH knowledge, attitudes, and behaviors, including age, working years, educational level, title, IPH training experience and so on [32, 33]. In addition, it has been suggested that knowledge, attention, environment and resources are the main influences on anesthesiologists’ hypothermia prevention practices [34]. Despite numerous influencing factors, existing research still suggests that nurses have insufficient knowledge and poor practical behavior in preventing hypothermia. Thus, to enhance the prevention techniques for Inadvertent Perioperative Hypothermia (IPH) and elevate the standard of perioperative care, it would be beneficial to investigate the knowledge, attitudes, and behaviors of nurses. Notably, there is a lack of studies on this topic in children’s tertiary specialized hospitals in China. Therefore, the outcomes of this study will contribute valuable insights into the knowledge, attitudes, and behaviors of surgical and anesthesia nurses concerning IPH prevention in China.

## Methods

### Study design

This study received approval from the Ethics Review Committee of the Children’s Hospital of Shanghai (2023R066-E01) and adhered to the principles outlined in the Declaration of Helsinki. A multicenter cross-sectional study was conducted from November 2023 to December 2023.

### Participants

A two-stage sampling method was employed. Firstly, the availability of participants was considered to achieve a homogeneous regional distribution of the samples. Four provinces (Hunan, Zhejiang, Anhui, and Shanghai) in the eastern, southern, and central areas of mainland China were selected using the purposive sampling method. Secondly, the convenience sampling method was utilized to select seven children’s tertiary specialized hospitals from the aforementioned areas. All participants provided signed informed consent and voluntarily participated in this study. The inclusion criteria were: (a) possession of a professional qualification certificate in the People’s Republic of China; (b) a minimum of 1 year of working experience as an operating room or anesthesia nurse; (c) no prior or current diagnosis of mental illness, drug or alcohol dependence; (d) basic computer skills; (e) informed consent to participate in the study. Nurse trainees, clinical interns, and those who failed to complete the survey were excluded.

### Measures

All tests were conducted in Mandarin Chinese.

#### Demographic variables

A self-made demographic questionnaire was utilized to collect participants characteristics, including gender, age, nurse classification, working years, educational level, position, title, number of IPH trainings, perioperative insulation process (Y/N), and familiarity with the 2023 Expert Consensus on Prevention and Treatment of Hypothermia in Perioperative Patients [35] (Y/N).

#### Knowledge, attitudes, and behaviors scale

In this study, we developed our own “Knowledge, attitudes, and behaviors scale” on inadvertent perioperative hypothermia prevention (IPH) by reviewing domestic and international literature, promulgated guidelines [35, 36] and expert panel review. Reliability and validity testing is an important step in evaluating the quality of a questionnaire, where Cronbach’s α is used to assess the reliability (consistency) and KMO (Kasier-Meyer-Olkin) value is used to evaluate the validity of the questionnaire. The internal consistency reliability of overall questionnaire was 0.888, of which knowledge, attitudes, and behaviors subscale were 0.740, 0.873, and 0.952, respectively. KMO value of validity analysis of overall questionnaire was 0.885, of which knowledge, attitudes, and behaviors subscale were 0.817, 0.877, and 0.948, respectively.

The knowledge section comprised a total of 22 items, covering concepts and mechanisms, risk factors, complications, temperature monitoring, and knowledge related to hypothermia prevention in current regulatory guidelines. Responses were in the form of true or false or unclear questions, with 1 point awarded for each correct answer and 0 for false or unclear responses. A higher score indicated better knowledge mastery. The attitudes section included 11 items, utilizing a Likert 5-point rating system: strongly agree (5 points), agree (4 points), neutral (3 points), disagree (2 points), strongly disagree (1 point). Reverse entry reverse scoring was applied, with a total score ranging from 11 to 55 points. The behaviors section consisted of 19 items, scored from 1 to 3 for “not achieving” to “fully achieving”, with a total score ranging from 19 to 57. A higher score reflected better implementation of insulation measures.

#### General self-efficacy scale (GSES)

The General Self-Efficacy Scale (GSES), developed by Jerusalem and Schwartz in 1979 [37], featured 10 items on a five-point ordinal scale ranging from 1 (“extremely inconsistent”) to 4 (“extremely consistent”), using the Likert 4-point rating system. Categorized into three levels—low level (< 20 points), medium level (20–30 points), and high level (> 30 points)—lower scores indicated lower self-efficacy. The Cronbach’s α was 0.87.

### Data collection

Nurses were requested to complete an anonymous online survey, facilitated through the “Questionnaire Star” platform and accessible via the link (www.wjx.cn). After obtaining permission, nursing managers from each hospital assisted in data collection by distributing the questionnaire link to nurses. The guide page clarified the survey’s purpose and completion requirements, ensuring anonymity. Participants provided informed consent before participating. All questionnaires were self-rated, with participants filling them out separately. Due to large number of items in this questionnaire and the involvement of professional knowledge, 5 min will be used as an indicator of the validity of the questionnaire responses. Answering time less than 5 min will be excluded.

### Statistical analysis

To assess the current knowledge, attitudes, and behaviors of pediatric surgical or anesthesia nurses regarding perioperative hypothermia protection and risk factors, various statistical analyses were conducted using SPSS 25.0 (IBM, Armonk, NY, USA). Shapiro-Wilk test was used for normality test. Descriptive statistics were used for count data (composition ratio [n (%)]) and measurement data (mean [standard deviation, SD] for normal distribution; median and quartiles [M (Q1, Q3)] for non-normal distribution). Group comparisons employed t-tests, analysis of variance (ANOVA), LSD (Least Significant Difference) analysis, as appropriate. Pearson correlation analysis explored relationships between self-efficacy, knowledge, attitudes, and behaviors. Multiple linear stepwise regression analysis identified factors influencing surgical or anesthesia nurses’ knowledge, attitudes, and behaviors regarding perioperative hypothermia protection. A *P*-value 

## Results

In total, 320 nurses were initially recruited, and after data cleaning, 292 were included in the analysis. Twenty participants were excluded due to providing a definitive answer in less than 5 min, and an additional eight had incomplete responses. The retention rate was 91.25%. Shapiro-Wilk test showed *P* < 0.05, combined with histogram effect, we concluded that data showed an approximate normal distribution.

### Participants’ demographics

292 nurses from Hunan, Zhejiang, Anhui, and Shanghai provinces in China were recruited for the study. Most participants were recruited from Shanghai (66.4%, 194/292), the least number of participants was recruited from Hunan (8.9%, 26/292). Age of participants ranged from 19 to 66 years, and the mean (SD) age was 32.49 (7.45) years. Their average years of working in operating room was 8.36 (7.08) years. The details of the study were shown in Table 1.

**Table 1:** Demographics of participants (*N* = 292)

Variables		*N* (%)
Region	Shanghai	194(66.4%)
	Zhejiang	44(15.1%)
	Hunan	26(8.9%)
	Anhui	28(9.6%)
Age	<25	46(15.8%)
	25–29	63(21.6%)
	30–34	78(26.7%)
	35–39	59(20.2%)
	≥ 40	46(15.8%)
Gender	Male	60(20.5%)
	Female	232(79.5%)
Classification	Surgical nurses	226(77.4%)
	Anesthesia nurses	66(22.6%)
Working years	<5	113(38.7%)
	5–9	67(22.9%)
	10–14	55(18.8%)
	15–19	28(9.6%)
	≥ 20	29(9.9%)
Educational level	Junior college	61(20.9%)
	Bachelor degree	218(74.7%)
	Master and above	13(4.5%)
Position	None	205(70.2%)
	Educational nurse	15(5.1%)
	Specialist nurse	60(20.5%)
	Head nurse	12(4.1%)
Title	Nurse	64(21.9%)
	Nurse practitioner	137(46.9%)
	Nurse in charge	81(27.7%)
	Deputy chief/chief nurse	10(3.4%)
No. of IPH training	0	42(14.4%)
	1	79(27.1%)
	2	50(17.1%)
	≥ 3	121(41.4%)
Perioperative insulation process	Yes, strictly enforced	210(71.9%)
	Yes, need to be further improved	69(23.6%)
	No	13(4.5%)
2023 Expert Consensus ^[35]^^	Yes	209(71.6%)
	No	83(28.4%)

^^^is 2023 Expert Consensus on Prevention and Treatment of Hypothermia in Perioperative Patients

### Knowledge, attitudes, and behaviors scale

The knowledge scale, scored out of 22, yielded results ranging from a minimum of 0 to a maximum of 21, with a mean score of 15.59 (SD = 3.28). The correct score percentages for individual questionnaire entries varied from 5.8 to 93.5%, with item 5 registering the lowest correct rate. Specific scores for each dimension of the knowledge scale are presented in Table 2. Full score ratio is the ratio of number of nurses who answered questions for each entry in dimensions with a full score to the total number of nurses who responded to each entry in dimension. The mean (SD) scores for attitudes and behaviors were 47.02 (8.05) and 52.48 (6.35), respectively. Total score of the knowledge, attitudes, and behaviors scale was 115.08 (12.61). Only correct rates of the knowledge scale were calculated, the total correct rates of knowledge, attitudes, and behaviors scale couldn’t be summarized. Table 3 displays the scores for each item on attitudes and behaviors scale, while items within the knowledge scale are represented as correct rates. In knowledge scale, correct rate is the ratio of number of nurses who answered each entry correctly to the total number of nurses.

**Table 2:** Each dimension of knowledge scale (*N* = 292)

Dimension	Full score	Mean (SD)	Full score ratio
Concepts and mechanisms	3	2.60(0.72)	71.58%
Risk factors	3	1.23(0.52)	1.03%
Complications	3	2.33(0.79)	49.32%
Warming knowledge	5	3.68(0.96)	9.93%
Temperature monitoring	8	5.75(1.71)	7.88%

**Table 3:** The score of knowledge, attitudes, and behaviors of IPH protection

Scale	Items	Score (M ± SD) /Correct rate
Knowledge	1.Perioperative hypothermia is defined as a core temperature	85.6%
	2.IPH is defined as a decrease in body temperature that occurs at any time during the perioperative period (especially intraoperatively) that is harmful to the organism, excluding therapeutic hypothermia (e.g., intentional lowering of the patient’s body temperature during cardiac surgery) or cooling of the patient in cases of malignant hyperthermia.	86.3%
	3.A 3-phase pattern of core temperature change in children undergoing surgery under general anesthesia: a period of rapid decrease-slowly decreasing period-plateau period.	88.7%
	4.Under general anesthesia, the body regulates changes in body temperature through autonomic and behavioral responses.	24.0%
	5.There are three major risk factors for the development of IPH: the application of anesthetic drugs, the hypothermic environment of the operating room, and the application of unheated fluids and/or blood products.	5.8%
	6.Hypothermia is more likely to occur in infants and young children, especially in premature and low-birth-weight children. This is mainly due to the incomplete development of the thermoregulatory center, thin subcutaneous fat, relatively large body surface area, and changes in ambient temperature, which can easily lead to IPH.	93.5%
	7.IPH can increase intraoperative bleeding in children.	81.5%
	8.IPH can increase the risk of surgical site infection in children.	88.4%
	9.IPH does not increase mortality in traumatized children.	63.0%
	10.Perioperative insulation devices are categorized as active warming and passive isolation. Active warming refers to application of devices such as inflatable heating blankets and infusion warming devices to maintain and increase body temperature; passive isolation refers to use of un-preheated surgical dressings or quilts to cover child’s skin to minimize heat loss.	88.4%
	11.A layer of mulch reduces heat loss by about 30%, and this improvement increases proportionately with more mulch.	20.2%
	12.Insulation blankets can be used to cover exposed body surface of child and the incubator can be used for transportation.	91.8%
	13.The safest and most effective method recommended by American Association of Operating Room Registered Nurses (AORN) to prevent IPH is the application of inflatable heating blankets.	87.7%
	14.When an inflatable heating blanket is used to keep child warm during perioperative period, the perforated side of the blanket should always be facing the child, not the unpunched side.	79.8%
	15.Intravenous fluids (except penicillin, vitamins, certain anesthetic drugs) or blood products should be warmed and preheated to 36–37 °C for infusion.	78.4%
	16.The gold standard for core temperature measurement is temperature monitoring by pulmonary artery cannulation.	66.8%
	17.According to Clinical Anesthesia Monitoring Guidelines of the Anesthesiology Branch of the Chinese Medical Association, the most common used methods of monitoring core body temperature during general anesthesia are esophageal and nasopharyngeal temperature monitoring.	80.8%
	18.According to National Center for Quality Control of Anesthesia Specialties’ Expert Consensus on Prevention and Treatment of Hypothermia in Perioperative Patients, the trend of child’s temperature should be monitored dynamically during perioperative period, and the measurement site of neonates can be selected from the back.	61.0%
	19.According to the Technical Guidelines for Prevention and Control of Surgical Site Infections, fluids such as sterile saline at a temperature of 37 °C should be used for surgical site irrigation.	88.4%
	20.The Code of Practice for the Management of Hospital Operating Departments does not yet address the need to guard against intraoperative hypothermia in children.	36.6%
	21.Intraoperative operating room temperature for adults should not fall below 21 °C.	76.4%
	22.Increase the ambient temperature appropriately, with the temperature in operating rooms where children’s surgeries are performed not lower than 23 °C.	86.3%
Attitudes	1. Hypothermia is inevitable in children undergoing surgery, which is normal and of no particular concern.	4.54 ± 0.85
	2. Intraoperative application of insulation does not affect the core temperature of child, but rather increases workload of nurse.	4.43 ± 0.99
	3. It does not matter whether child develops hypothermia or not as long as the surgery can be performed successfully.	4.56 ± 0.89
	4. The occurrence of chills in children during the perioperative period is primarily an effect of anesthetic drugs and is not related to whether operating room nurses keep them warm.	4.45 ± 0.95
	5. The person responsible for the management of child’s temperature during the perioperative period is anesthesiologist; operating room nurses are only responsible for cooperating with the surgery.	4.39 ± 1.00
	6. Unplanned perioperative hypothermia can lead to a variety of complications that can seriously affect the outcome of surgical treatment, so operating room nurses should be proactive in applying a variety of warming measures for children.	4.06 ± 1.35
	7. Active intraoperative use of various insulation measures can effectively prevent occurrence of unplanned perioperative hypothermia and chills.	4.12 ± 1.30
	8. Operating room nurses play an important role in preventing occurrence of unplanned perioperative hypothermia.	4.18 ± 1.18
	9. Be able to speak up and correct others when you see them not doing a good job of perioperative insulation.	4.06 ± 1.17
	10. As an operating room nurse I would feel guilty or self-conscious if hypothermia or chills occurred in a child as a result of not actively applying warming measures.	4.01 ± 1.15
	11. There is a need to strengthen the training of operating room nurses in perioperative unplanned hypothermia and its protection and other related knowledge.	4.19 ± 1.15
	Overall	47.02 ± 8.05
Behaviors	1. When admitting children to operating room during cold season, ask them to wear good clothing or cover surgical trolley with a quilt to minimize skin exposure.	2.83 ± 0.39
	2. The temperature of operating room was adjusted to the normal range 1 h before the child was admitted to operating room, so as to prevent the child from feeling uncomfortable with temperature after entering the room.	2.81 ± 0.42
	3. Initiate communication with child upon entry into the operating room and inquire about child’s temperature comfort level.	2.70 ± 0.49
	4. The child is assessed for risk of unplanned perioperative hypothermia, while appropriate insulation measures are communicated and collaboratively developed with members of the surgical team.	2.75 ± 0.46
	5. When children enter the operating room to take off their gowns, cover them with a quilt, take off their pants first, then their tops, and minimize the amount of time their skin is exposed.	2.75 ± 0.46
	6. Once the child is lying on surgical bed, the cover should be fully unfolded to cover whole body, with special attention to keeping the feet and shoulders warm.	2.79 ± 0.43
	7. The child was carefully inspected after completion of surgical position and exposed limbs were kept warm with small quilts and/or cloth sheets.	2.81 ± 0.42
	8. When disinfecting preoperatively, minimize the area of exposed skin while meeting the criteria for the scope of disinfection.	2.80 ± 0.42
	9. Try to avoid soaking the surgical sheets with antiseptic solution during preoperative sterilization.	2.74 ± 0.48
	10. The temperature of operating room was maintained at ≥ 23 °C during pediatric surgery, and temperature of the operating room was adjusted appropriately according to the different periods of surgery.	2.71 ± 0.51
	11. During surgery, except in special cases, the surgical site is flushed with fluid preheated to 37 °C in a thermostat.	2.72 ± 0.49
	12. When large volumes of fluids and/or blood are transfused to expand blood volume during surgery, they are infused using a heated infusion/blood transfusion device.	2.78 ± 0.44
	13. Assist anesthesiologist in continuously monitoring changes in the patient’s core temperature during surgery and take care to assess the child for signs of hypothermia (e.g., assess the patient for cold extremity endings).	2.71 ± 0.51
	14. Correct and safe perioperative use of the various holding instruments and equipment available in the department.	2.83 ± 0.40
	15. Remove the soaked surgical dressing and cover the child with a pre-warmed quilt promptly after the procedure.	2.77 ± 0.45
	16. After surgery, the child was sent back to the ward when his core temperature was not lower than 36 °C.	2.75 ± 0.46
	17. Keep child warm and avoid exposing skin on the way back to the ward after operation.	2.84 ± 0.39
	18. During postoperative transport of child from surgical trolley to regular bed, ensure that the surface of patient’s skin is covered with a quilt to avoid direct exposure of skin to the air.	2.83 ± 0.38
	19. At the return visit 1–3 days postoperatively, the children were asked about their comfort level in operating room and their body temperature upon return to the ward for better caloric management.	2.58 ± 0.61
	Overall	52.48 ± 6.35

*M* is mean, *SD* is standard deviation

### Self-efficacy

General self-efficacy was classified into low, medium, and high levels. In this study, nurses’ self-efficacy ranged from 11 to 40 scores, with a mean (SD) score of 29.33 (4.53). 3 nurses (1.0%) demonstrated a low level, 217 nurses (74.3%) demonstrated a moderate level, and 72 nurses (24.7%) demonstrated a high level of self-efficacy.

### Factors influencing nurses’ knowledge, attitudes, and behaviors in IPH protection

Table 4 illustrates the between-group differences in knowledge, attitudes, and behavior scores based on region and demographic variables. Results from one-way analysis of variance indicated that different regions, working years, educational level, number of IPH trainings, and the implementation of a perioperative insulation process were significant risk factors for knowledge scores in IPH protection (*P* < 0.05). Statistically significant differences in attitudes scores related to IPH protection were observed among participants of different regions, genders, ages, working years, titles, and number of IPH trainings (*P* < 0.05). Regarding behaviors, influencing factors included region, the number of IPH trainings, the existence of a perioperative insulation process, awareness of the 2023 Expert Consensus on Prevention and Treatment of Hypothermia in Perioperative Patients, and self-efficacy (*P* < 0.05).

The LSD two-by-two comparison results presented in Table 4 revealed that nurses in Anhui province, more working years (within 20 years), higher educational levels, increased IPH trainings, and those who had a perioperative insulation process and rigorously implemented it achieved higher knowledge scores. Additionally, nurses in Anhui province, female participants, older individuals, those with more working years (within 20 years), higher titles, and increased IPH trainings attained higher scores in attitudes. Regarding behaviors, nurses in Anhui province, more IPH trainings, an established perioperative insulation process that was strictly enforced, awareness of the 2023 Expert Consensus on Prevention and Treatment of Hypothermia in Perioperative Patients, and higher self-efficacy scores received higher behavior scores.

**Table 4:** Results of univariate analysis of IPH protection knowledge, attitudes, and behaviors scores (*N* = 292)

Variables		Knowledge(M ± SD)	Attitudes(M ± SD)	Behaviors(M ± SD)	LSD
Region	(1) Shanghai	15.24 ± 3.62	46.50 ± 8.07	52.72 ± 6.35	
	(2) Zhejiang	16.02 ± 2.41	48.84 ± 7.42	49.52 ± 7.89	
	(3) Hunan	15.88 ± 2.64	43.42 ± 8.24	53.81 ± 4.07	
	(4) Anhui	17.07 ± 1.74	51.04 ± 6.77	54.18 ± 3.55	
*t*/*F*		3.014	5.288	4.471	
*P*		0.030 ^&^	0.001 ^#^	0.004 ^$^	^&^(1)(4)^*^ ^#^(1)(4)^*^ (2)(3)^*^ (3)(4)^*^ ^$^(1)(2)^*^(2)(3)^*^ (2)(4)^*^
Gender	Male	15.35 ± 3.21	44.82 ± 8.82	53.23 ± 6.06	
	Female	15.66 ± 3.30	47.59 ± 7.76	52.28 ± 6.42	
*t*/*F*		−0.641	−2.394	1.036	——
*P*		0.522	0.017	0.301	
Age	(1) <25	14.46 ± 4.11	42.43 ± 8.54	53.39 ± 5.84	
	(2) 25–29	15.54 ± 3.65	46.90 ± 7.50	52.16 ± 6.07	
	(3) 30–34	16.14 ± 2.53	49.23 ± 7.53	52.53 ± 6.98	
	(4) 35–39	15.85 ± 3.16	47.61 ± 7.92	53.10 ± 5.70	
	(5) ≥ 40	15.54 ± 2.94	47.24 ± 7.78	51.11 ± 6.89	
*t*/*F*		2.047	5.625	0.954	^#^(1)(2)^**^(1)(3)^**^ (1)(4)^**^(1)(5)^**^
*P*		0.088	< 0.001 ^#^	0.433	
Classification	Surgical nurses	15.70 ± 3.37	47.27 ± 7.83	52.80 ± 6.08	
	Anesthesia nurses	15.23 ± 2.96	46.15 ± 8.77	51.36 ± 7.14	
*t*/*F*		1.028	0.993	1.621	——
*P*		0.305	0.322	0.106	
Working years	(1) <5	14.68 ± 4.03	44.41 ± 8.34	51.71 ± 7.19	
	(2) 5–9	16.22 ± 2.57	49.45 ± 7.24	52.43 ± 6.50	
	(3) 10–14	16.24 ± 2.49	48.04 ± 7.76	53.75 ± 4.85	
	(4) 15–19	16.25 ± 2.20	49.21 ± 6.52	53.61 ± 4.55	
	(5) ≥ 20	15.83 ± 3.01	47.52 ± 8.14	52.07 ± 6.40	
*t*/*F*		3.784	5.595	1.218	^&^(1)(2)^**^(1)(3)^**^(1)(4)^* #^(1)(2)^**^(1)(3)^**^ (1)(4)^**^
*P*		0.005 ^&^	< 0.001 ^#^	0.303	
Educational level	(1) Junior college	14.65 ± 4.34	44.98 ± 8.28	52.31 ± 6.52	
	(2) Bachelor degree	15.72 ± 2.92	47.47 ± 7.88	52.64 ± 6.26	
	(3) Master and above	17.85 ± 1.52	49.00 ± 8.77	50.54 ± 7.27	
*t*/*F*		5.907	2.712	0.694	^&^(1)(2)^*^(1)(3)^**^
*P*		0.003 ^&^	0.068	0.500	
Position	(1) None	15.68 ± 3.26	47.39 ± 7.85	52.52 ± 6.57	
	(2) Educational nurse	14.80 ± 5.00	44.47 ± 8.63	52.20 ± 6.53	
	(3) Specialist nurse	15.28 ± 3.11	46.03 ± 8.34	53.12 ± 5.39	
	(4) Head nurse	16.58 ± 1.31	48.67 ± 9.20	48.92 ± 6.40	
*t*/*F*		0.884	1.120	1.478	——
*P*		0.45	0.341	0.221	
Title	(1) Nurse	15.25 ± 3.32	43.22 ± 8.47	54.23 ± 5.05	
	(2) Nurse practitioner	15.43 ± 3.68	48.39 ± 7.34	52.11 ± 6.72	
	(3) Nurse in charge	16.16 ± 2.29	47.46 ± 8.23	52.01 ± 6.39	
	(4) Deputy chief/chief nurse	15.40 ± 3.98	49.00 ± 6.43	50.00 ± 6.88	
*t*/*F*		1.166	6.724	2.473	^#^(1)(2)^**^(1)(3)^**^ (1)(4)^*^
*P*		0.323	< 0.001 ^#^	0.062	
No. of IPH training	(1) 0	14.26 ± 4.93	45.67 ± 9.31	49.36 ± 8.52	
	(2) 1	14.51 ± 3.60	44.44 ± 8.05	52.47 ± 6.56	
	(3) 2	16.44 ± 2.37	45.96 ± 8.12	52.46 ± 6.16	
	(4) ≥ 3	16.41 ± 2.17	49.60 ± 6.81	53.57 ± 5.00	
*t*/*F*		9.601	8.080	4.747	^&^(1)(3)^**^(1)(4)^**^ (2)(3)^**^(2)(4)^** #^(1)(4)^**^(2)(4)^**^ (3)(4)^**^ ^$^(1)(2)^**^(1)(3)^*^ (1)(4)^**^
*P*		< 0.001 ^&^	< 0.001 ^#^	0.003 ^$^	
Perioperative insulation process	(1) Yes, strictly enforced	15.99 ± 2.87	46.95 ± 8.59	54.23 ± 4.83	
	(2) Yes, need to be further improved	14.59 ± 3.65	47.58 ± 6.30	48.09 ± 7.38	
	(3) No	14.46 ± 5.68	45.08 ± 7.60	47.46 ± 8.46	
*t*/*F*		5.685	0.551	35.202	^&^(1)(2)^** $^(1)(2)^**^(1)(3)^**^
*P*		0.004 ^&^	0.577	< 0.001 ^$^	
2023 Expert Consensus ^[35]^^	Yes	15.80 ± 3.21	46.55 ± 8.39	53.41 ± 5.56	
	No	15.06 ± 3.42	48.19 ± 7.04	50.12 ± 7.54	
*t*/*F*		1.752	−1.576	4.099	——
*P*		0.081	0.116		
Self-efficacy	(1) Low	14.67 ± 4.04	42.33 ± 9.02	50.67 ± 6.03	
	(2) Medium	15.47 ± 3.53	46.52 ± 7.97	51.91 ± 6.60	
	(3) High	16.00 ± 2.33	48.69 ± 8.11	54.26 ± 5.23	
*t*/*F*		0.824	2.499	3.917	^$^(2)(3)^**^
*P*		0.440	0.084	0.021 ^$^	

*LSD* is least significant difference

^**^P < 0.01, ^*^P < 0.05

^ is 2023 Expert Consensus on Prevention and Treatment of Hypothermia in Perioperative Patients

Multiple linear regression analysis was conducted to examine the relationship between various variables as independent variables and dimensions of knowledge, attitudes, and behaviors as dependent variables (Table 5). The analysis of covariance revealed VIF value was < 10, thus, there was no multicollinearity between independent variables. The results indicated that educational level, number of IPH trainings, and the presence of a perioperative insulation process were significant predictors, entering the regression model (*F* = 9.235, *P* < 0.001), explaining 12.4% of the variance in knowledge scores (adjusted *R*^2^ = 0.124). The standardized regression coefficients (Beta) revealed that an enhanced educational level and an increased number of IPH trainings were associated with improved knowledge among nurses. Additionally, knowledge scores decreased when the hospital lacked a perioperative insulation process.

In a separate analysis, the number of IPH trainings entered the regression model (*F* = 4.409, *P* < 0.001), explaining 6.6% of the variance in knowledge scores (adjusted *R*^2^ = 0.066). The standardized regression coefficients (Beta) indicated that an increased number of IPH trainings was a significant factor associated with improved nurses’ attitudes.

Concerning behavior, the regression model included the number of IPH trainings, perioperative insulation process, and self-efficacy (*F* = 17.642, *P* < 0.001), explaining 22.2% of the variance in behavior scores (adjusted *R*^2^ = 0.222). The standardized regression coefficients (Beta) demonstrated that an increased number of IPH trainings and higher self-efficacy were associated with improved behavior among nurses. Similarly, behavior scores decreased when the hospital lacked a perioperative insulation process.

**Table 5:** Multiple linear regression analysis for influencing factors of nurses’ knowledge, attitudes, and behaviors

Dimension	Item	b	SE-b	Beta	t	*p*	95% CI	
							Lower	Upper
Knowledge	Constant	12.714	0.937		13.576	<0.001	10.871	14.558
	Region	0.263	0.187	0.080	1.409	0.160	−0.104	0.631
	Working years	0.209	0.144	0.085	1.444	0.150	−0.076	0.493
	Educational level	0.882	0.387	0.128	2.278	0.023	0.120	1.645
	No. of IPH training	0.596	0.175	0.203	3.408	0.001	0.252	0.941
	Perioperative insulation process	−1.017	0.329	−0.172	−3.091	0.002	−1.665	−0.369
Attitudes	Constant	36.452	2.494		14.617	<0.001	31.543	41.360
	Region	0.460	0.504	0.057	0.913	0.362	−0.532	1.453
	Gender	2.175	1.205	0.109	1.805	0.072	−0.197	4.546
	Age	0.349	0.570	0.056	0.612	0.541	−0.773	1.472
	Working years	0.140	0.527	0.023	0.266	0.790	−0.897	1.178
	Title	0.241	0.807	0.024	0.299	0.765	−1.347	1.829
	No. of IPH training	1.415	0.446	0.196	3.175	0.002	0.538	2.292
Behaviors	Constant	51.165	2.771		18.461	<0.001	45.710	56.620
	Region	−0.166	0.339	−0.026	−0.491	0.624	−0.833	0.500
	No. of IPH training	0.766	0.309	0.134	2.476	0.014	0.157	1.375
	Perioperative insulation process	−4.159	0.629	−0.364	−6.612	<0.001	−5.398	−2.921
	2023 Expert Consensus^[2]^^	−1.090	0.775	−0.078	−1.406	0.161	−2.616	0.436
	Self-efficacy	0.215	0.075	0.153	2.851	0.005	0.067	0.363

*b* is the unstandardized regression coefficient, *SE-b* is the standard error, *Beta* is the standardized regression coefficient, *CI* is the confidence coefficient

*^*is 2023 Expert Consensus on Prevention and Treatment of Hypothermia in Perioperative Patients

### Correlation analysis of major variables

Pearson correlation analysis showed that self-efficacy was positively related to knowledge (*r* = 0.137, *P* < 0.05), attitudes (*r* = 0.115, *P* < 0.05), and behaviors (*r* = 0.258, *P* < 0.01). In addition, knowledge was positively related to attitudes (*r* = 0.262, *P* < 0.01), and behaviors (*r* = 0.322, *P* < 0.01). Similarly, attitudes were positively related to behaviors (*r* = 0.153, *P* < 0.01) (Table 6).

**Table 6:** Correlation analysis of major variables

Variables	1	2	3	4
1.Self-efficacy	—			
2.Knowledge	0.137^*^	—		
3.Attitudes	0.115^*^	0.262^**^	—	
4.Behaviors	0.258^**^	0.322^**^	0.153^**^	—

^*^P < 0.05, ^**^P < 0.01

## Discussion

This multicenter cross-sectional study was conducted in seven tertiary specialized children’s hospitals from four provinces in the east, south, and central areas of mainland China. This study represents the first exploration of the current situation regarding the knowledge, attitudes, and behaviors of surgical nurses and nurse anesthetists concerning inadvertent perioperative hypothermia (IPH) prevention in children’s tertiary specialized hospitals in China, as well as an investigation into its influencing factors.

### The current situation of nurses’ knowledge, attitudes, and behaviors

In the prevailing context of robustly advocating “patient safety,” IPH, as a significant safety concern for surgical patients, has garnered the attention of operating room nursing staff. The results of this study revealed that the knowledge dimension of surgical nurses and anesthesia nurses in Chinese tertiary specialized children’s hospitals was at an intermediate level, with a mean score of 15.59 (3.28), which was higher than Chen’s research (13.56 ± 1.96) in Heilongjiang province, China [38]. Out of 292 surveyed nurses, 60.27% achieved a passing score (score/total > 0.6), and only 22.94% obtained an excellent score (score/total > 0.8), surpassing results reported by Jallow et al. [29]. The analysis of total scores for each dimension indicated that while nurses demonstrated a good understanding of the concepts and mechanisms of hypothermia, there is room for improvement in their knowledge of risk factors, perioperative temperature monitoring, warming techniques, and associated complications. These findings suggest a potential disregard for IPH literature and ignorance of IPH recommendations among nurses. The study’s results align with earlier research in this area [39, 40]. Notably, nearly half of the nurses (41.5%) attended ≤ 1 IPH training session, which may have contributed to this result. Majority of nurses (90.1%) acknowledge the need for IPH training, validating our assessment. At present, scholars at home and abroad are increasingly paying attention to the management of IPH by healthcare personnel [41]. Although many guidelines for preventing IPH have been published, IPH remains a problem [35, 42]. This outcome highlights the necessity of updating knowledge by organizing education programs focused on IPH in the perioperative nursing environment for children and further integrating practice into clinical protocols.

Following the Knowledge-Attitude-Behavior paradigm, knowledge serves as the foundation for behavior change, while attitude acts as the driving force [43]. The attitude dimension received an average score of 47.02 (8.05), indicating that nurses in this multidisciplinary study held positive attitudes toward IPH prevention, akin to the findings of Chen et al. [38]. The study suggests that training interventions might enhance IPH knowledge, improve attitudes toward IPH prevention, and increase compliance with preventative procedures among operating room nurses [44]. Similar improvements were noted in studies on palliative care knowledge and attitudes following training [45], as well as in the application of physical restraints in the intensive care unit [46]. Training and education in the workplace were found to lower the incidence rate of IPH in the neonatal intensive care unit (NICU) [47]. Scholars have pointed out that training programs guided by adult learning theory [38], implementing performance and ability based health training measures [48] can significantly improve scores of operating room nurses in IPH prevention related knowledge, improve their attitudes towards perioperative hypothermia prevention, and enhance their compliance with IPH prevention interventions.

Regarding nurses’ behaviors in IPH prevention, the results showed a mean score of 52.48 (6.35). Most participants selected the “completely done” response for the statements, which contrasts with a qualitative study by Honkavuo et al. [31]. Two items with the lowest scores pertained to nurses actively communicating with children and inquiring about their temperature comfort level after entering the operating room, as well as assessing the comfort level and temperature of children post-surgery during the 1–3 days follow-up. These findings deviate from the perioperative hypothermia prevention guidelines published by AORN [47] and the National Institute for Health and Care Excellence (NICE) [49]. The discrepancy between nurses’ self-evaluation behavior and the guidelines’ requirements fundamentally constitute a systemic misalignment within humanistic care practices within an efficiency-driven healthcare system, particularly evident in the communication and ongoing temperature regulation for specific pediatric cohorts.

### The influencing factors of nurses’ knowledge, attitudes, and behaviors

It had been reported that educational level was in the risk group for IPH knowledge dimension; whether there was a perioperative insulation process was the risk factor for knowledge and behavior dimensions; No. of IPH training was the risk factor for all three knowledge, attitudes, and behaviors dimensions; self-efficacy was the risk factor for behavior dimension.

Perioperative hypothermia prevention requires multidisciplinary communication and collaboration. In addition to physicians, nurses should be knowledgeable about preoperative, intraoperative, and postoperative hypothermia prevention. Educational level reflects the ability to learning of nursing staff. The higher the level of education, the better the knowledge structure of nurses. According to this study, nurses with a bachelor’s and master’s degree had a higher likelihood of being well-versed in preventing hypothermia than those with only a junior college. Studies done in Brazil [50] and Iran [51] provide evidence in favor of this conclusion. This suggests that nurses need to enhance their scientific knowledge and, consequently, raise the standard of patient care, educational interventions are essential [50]. For example, tailor-made training courses for nurses with different educational backgrounds; combining intelligent tools and reward and punishment mechanisms for IPH knowledge assessment; integrate IPH prevention knowledge into daily operational processes, et al.

Moreover, this might be clarified by the idea that nurses with greater education would have taken different training on avoiding hypothermia. When compared nurses who did not get training on hypothermia prevention to those who did, knowledge, attitudes, and behaviors of the former group was superior. Other researches, such as conducted in America [52], Iran [51], and Turkey [53] confirmed this results. This makes sense that increasing the caliber of patient care is mostly dependent in training.

Regarding nurses’ knowledge and behavior of hypothermia prevention, this study identified that equipped with perioperative insulation process had better knowledge and behavior than those did not have a hypothermia perioperative insulation process, which was similar with results of Woretaw et al. [54]. When hospital with a complete perioperative insulation process, it is beneficial to standardize the workflow of nurses and prevent the occurrence of hypothermia. Furthermore, self-efficacy served as a protective factor against nurses’ behavior, just like workplace misconduct [55]. The premise of self-efficacy theory is that people’s perceptions of their skills and abilities influence their behavior and serve as the foundation for their activities. Self-efficacy of nurses improves the standard of patient care. According to research evaluation, there is a strong correlation between job performance and self-efficacy [56]. A sense of high self-efficacy motivates individuals to set challenging goals and persist in refining strategies, thereby significantly enhancing performance. In turn, performance achievements, bolstered by successful experiences and social persuasion, reinforce the sense of efficacy, creating a self-reinforcing loop of self-regulation. A nurse’s clinical practice may be predicted using self-efficacy assessment [57]. Because they are better at solving difficulties, those who have high self-efficacy can solve problems more quickly. When issues and challenging circumstances emerge, they concentrate on their capacity to resolve the issue and seek for a fresh approach.

### Recommendations

Perioperative temperature management is an important initiative that promotes safety and comfort of children as an inevitable trend to improve overall quality of nursing. The special physiological characteristics of children determine that their body temperature is more susceptible to environmental changes [58]. If not properly insulated, IPH may be higher than that seen in adults. Passive insulation is now widely used for perioperative temperature management. However, a single layer of coverage only reduces skin heat loss by about 30%, and even more coverage is difficult to reduce patient’s heat loss to 50% [59]. In contrast, active insulation measures are more effective. Active insulation measures including air heating blankets, heating infusers, and thermostats have been partially applied to perioperative temperature management in children with good results [60]. In addition, it is suggested that operating room should be equipped with different temperature monitoring instruments according to different types of surgery. Operating room nurses or anesthesia nurses should be instructed to increase or decrease application of thermal insulation measures at the appropriate time. Notably, it is critical to develop a standardized perioperative insulation process for children. Children’s specialty hospitals can develop feasible holding procedures based on standardized guidance from healthcare professionals with reference to existing research results and domestic and international guidelines. Finally, it is recommended that nursing administrators incorporate the incidence of hypothermia into a monitoring system of sensitive indicators, analyze causes, and make continuous improvements to enhance the quality of care.

### Limitations

This study had several limitations. Firstly, the generalizability was limited since we could only choose four provinces from China’s central, eastern, and southern regions due to time and budgetary constraints. Secondly, a total of seven hospitals with operating room or anesthesia nurses were included in this study, out of which six hospitals belonged to children’s specialty hospitals, only one hospital belonged to a comprehensive hospital, and secondary hospitals were not included. Thirdly, the cross-sectional study used convenience sampling to select research subjects, thus, the determination of samples is arbitrary and cannot represent the population. Fourthly, the absence of a pre-study power calculation may limit the generalizability of statistical inferences. Given the exploratory design, this study prioritizes identifying potential associations over rigorous hypothesis testing. The exploratory analysis involved multiple statistical tests without formal adjustment for multiplicity. While this approach increases sensitivity to detect potential associations, it also elevates Type I error risk. Significant results (especially near-threshold *p*-values) should be interpreted cautiously and require independent validation.

## Conclusion

This study investigated the current situation of anesthesia and operating room nurses’ knowledge, attitudes, and behaviors of IPH prevention and its influencing factors, which could provide a reference for nursing managers to develop standardized perioperative insulation processes. Results indicated that nurses’ knowledge of IPH prevention was at an intermediate level. Educational level was in the risk group for knowledge dimension; whether there was a perioperative insulation process was the risk factor for knowledge and behavior dimensions; No. of IPH training was the risk factor for all three knowledge, attitudes, and behaviors dimensions; self-efficacy was in the risk factor for behavior dimension. This study has demonstrated that nursing managers should increase the frequency of IPH training and develop standardized perioperative insulation process. Nurses should also enhance their theoretical knowledge learning, improve educational level and self-efficacy.

## Data Availability

Data is provided within the manuscript files.

## Abbreviations

ANOVA: Analysis of Variance
AORN: American Peri-Operative Registered Nurses
GSES: General Self-efficacy Scale
IPH: Inadvertent Perioperative Hypothermia
KMO: Kasier-Meyer-Olkin
LSD: Least Significant Difference
M: Mean
NICE: National Institute for Health and Care Excellence
NICU: Neonatal Intensive Care Unit
No.: Number
SD: Standard Deviation
VIF: Variance Inflation Factor
